# Metabolomics and network pharmacology-based identification of phenolic acids in *Polygonatum kingianum* var. *grandifolium* rhizomes as anti-cancer/Tumor active ingredients

**DOI:** 10.1371/journal.pone.0315857

**Published:** 2024-12-17

**Authors:** Xiaolin Wan, Lingjun Cui, Qiang Xiao

**Affiliations:** Hubei Key Laboratory of Biological Resources Protection and Utilization (Hubei Minzu University), Enshi, China; Dr Rammanohar Lohia Avadh University, INDIA

## Abstract

Broadly targeted metabolomics techniques were used to identify phenolic acid compounds in *Polygonatum kingianum* var. *grandifolium* (PKVG) rhizomes and retrieve anti-cancer/tumor active substance bases from them. We identified potential drug targets by constructing Venn diagrams of compound and disease targets. Further, KEGG pathway analysis was performed to reveal the relevant pathways for anti-cancer/tumor activity of PKVG. Finally, we performed molecular docking to determine whether the identified proteins were targets of phenolic acid compounds from PKVG rhizome parts. The study’s results revealed 71 phenolic acid compounds in PKVG rhizomes. Among them, three active ingredients and 42 corresponding targets were closely related to the anticancer/tumor activities of PKVG rhizome site phenolic acids. We identified two essential compounds and eight important targets by constructing the compound-target pathway network. 2 essential compounds were androsin and chlorogenic acid; 8 key targets were MAPK1, EGFR, PRKCA, MAPK10, GSK3B, CASP3, CASP8, and MMP9. The analysis of the KEGG pathway identified 42 anti-cancer/tumor-related pathways. In order of degree, we performed molecular docking on two essential compounds and the top 4 targets, MAPK1, EGFR, PRKCA, and MAPK10, to further validate the network pharmacology screening results. The molecular docking results were consistent with the network pharmacology results. Therefore, we suggest that the phenolic acids in PKVG rhizomes may exert anti-cancer/tumor activity through a multi-component, multi-target, and multi-channel mechanism of action.

## Introduction

The "Ming Yi Bie Lu" first published the *Polygonatum*, a perennial herb of the *Liliaceae* with the same origins as food and medicine. The 2020 edition of the Chinese Pharmacopoeia lists the Chinese medicine *Polygonatum*’s basal plants as *Polygonatum sibiritum* Red, *Polygonatum kingianum* coll.et Hemsl and *Polygonatum cyrtonema* Hua. The world distributes *Polygonatum*, including in China, Japan, Korea, India, Russia, Europe, and North America [1, 2]. *Polygonatum kingianum* var. *grandifolium* (PKVG), a variant of *Polygonatum kingianum* coll.et Hemsl, have garnered attention due to their large production volume and frequent use in folk medicine as a source of *Polygonatum* herbal medicine [3]. Previous studies have found that *Polygonatum* contain polysaccharides, saponins, flavonoids, lignans, amino acids, phenolic acids, alkaloids, and a variety of trace elements [4]. These compounds have demonstrated significant anti-fatigue, anti-aging, metabolic modulation, immunomodulation, anti-inflammatory, neuroprotective, anti-diabetic, and anti-cancer effects [5]. These compounds and pharmacological effects emphasize *Polygonatum*’s potential.

Traditional Chinese medicines’ therapeutic effects often stem from the synergistic effects of their multiple components, so it is critical to explore the role of their chemical components in depth. The key to achieving this research goal is constructing a scientific and efficient analytical tool. Broadly targeted metabolomics analysis is a new metabolomics approach. It combines the advantages of targeted metabolomics (high sensitivity) and non-targeted metabolomics (high throughput). It is distinguished by high throughput, ultra-sensitivity, and comprehensive coverage of metabolites, allowing it to analyze thousands of metabolites in plant samples in a single step, qualitatively and quantitatively [6]. Many fields use broadly targeted metabolomics technology, including agriculture, medicine, and food science. This study used broadly targeted metabolomics technology for rapid qualitative and quantitative analysis of chemical constituents in PKVG rhizomes. The study’s results can provide a reference for the quality control of PKVG rhizomes and a scientific basis for the study of the medicinal efficacy of PKVG rhizomes.

Phenolic acids are one of the most prevalent classes of bioactive chemicals of plant origin. They are found in large quantities in foods such as berries [7], nuts [8], and whole grains [9]. Phenolic acids have been reported to reduce the adhesion of various microbial cells, ultimately reducing the etiology of disease [10]. Other studies have shown that phenolic acids have received widespread attention for their potential antioxidant, cardioprotective, anti-inflammatory, anti-atherosclerotic, immunomodulatory, anti-allergic, anti-thrombolytic, antimicrobial, antitumor, anti-obesity, anticancer, and antidiabetic properties [11–13]. Currently, cancer has been recognized as the second-leading cause of death worldwide. Conventional methods for the treatment of cancer include chemotherapy or radiotherapy. However, these methods are usually associated with various side effects and many drawbacks in clinical practice [14]. With the rapid development of bioinformatics technology, cyber pharmacology has become a powerful tool for exploring the mechanisms of action of herbal medicines. It provides a systematic understanding of drug action and disease complexity and models constructed based on cyber pharmacology help to elucidate the role of herbal medicines in specific diseases [15].

Therefore, the present study combined metabolomics and network pharmacology to elucidate the anticancer/antitumor mechanism of phenolic acids in PKVG rhizomes. It provides an important basis for the study of new anticancer/antitumor drugs.

## Materials and methods

### Plant materials

The test sample was a three-year-old PKVG rhizome. We planted the PKVG at the test site of the College of Forestry and Horticulture, Hubei Minzu University. The bottom soil consisted of vermiculite and peat soil (8:1 ratio). We collected three biological replicates of PKVG rhizomes. We chopped, mixed, and loaded the sampled PKVG rhizomes into sterile centrifuge tubes, snap-frozen them in liquid nitrogen, and stored them at -80°C until we needed them.

### Sample preparation and extraction

Samples were prepared and extracted using methods provided by Metware Biotechnology Ltd. (Wuhan, China). The data acquisition instrumentation system mainly consisted of ultra-performance liquid chromatography (UPLC) (SHIMADZU Nexera X2) and tandem mass spectrometry (MS/MS) (Applied Biosystems 4500 QTRAP).

Liquid phase conditions: (i) column: Agilent SB-C18 1.8 μm, 2.1 mm * 100 mm; (ii) mobile phases: ultra-pure water (with 0.1% formic acid added) for phase A and acetonitrile (with 0.1% formic acid added) for phase B; (iii) the proportion of B-phase was 5% for wash 0.00 min, and then the proportion of B-phase was increased linearly to 95% within 9.00 min and was maintained at 95% for 1 min; from 10.00 to 11.10 min, the B-phase ratio decreased to 5% and equilibrated at 5% for 14 min; (iv) flow rate: 0.35 mL/min; column temperature: 40°C; injection volume: 4 μL. UPLC-MS/MS was carried out by Metware Biotechnology Ltd. (Wuhan, China). Metabolomics data were obtained in electrospray ionization negative (ESI-) and positive (ESIIC) modes. The ion spray voltage was -4500 V for ESI- and 5500 V for ESIC; the ion source gas I (GSI), gas II (GSII), and curtain gas (CUR) were set to 50, 60, and 25 psi, respectively; the collision-induced ionization parameter was set to high; and the electrospray ionization source (ESI) temperature was 550°C.

### Qualitative and quantitative metabolite analysis

Raw data were initially converted to mzXML format using Proteo Wizard. Subsequently, peak extraction, alignment, and retention time correction were performed using XCMS software. To ensure data quality, peaks with a missing rate exceeding 50% in any sample group were excluded, and missing values were imputed through the KNN method. The peak areas were further adjusted using the SVR method. Metabolites were identified by matching the processed peaks to the proprietary database of Metware Biotechnology Company (Wuhan, China), as well as public and predictive libraries, following the MetDNA approach. Only metabolites with a composite identification score ≥0.5 and a coefficient of variation (CV) <0.3 in QC samples were retained for subsequent analysis. Positive and negative ion modes were integrated to retain compounds with the highest identification confidence and the lowest CV values, generating a comprehensive data file for all samples. Metabolites were characterized using both primary and secondary MS data, and their relative abundance across samples was determined based on the chromatographic peak areas.

In order to compare the differences in the content of each metabolite among all the detected metabolites in different samples, based on the information of metabolite retention time and peak shape, we corrected the mass spectrometry peaks of each metabolite detected in different samples to ensure the accuracy of qualitative and quantitative.

### Data collection on active components and targets of phenolic acid compounds from PKVG rhizomes

We used the TCMSP database (https://old.tcmsp-e.com/tcmsp.php) to search for PKVG rhizome phenolic acid analog drug constituents. OB ≥ 10% and DL ≥ 0.1 [16] were used as the screening conditions to obtain the active ingredients of PKVG rhizome phenolic acid analogs and their corresponding targets, and the UniProt database (https://www.uniprot.org/) was used for canonical target naming. Finally, we used Cytoscape 3.9.1 software to make a network diagram of the active ingredients and targets of PKVG rhizome phenolic acid analogs.

### Cancer/tumor target information collection

In the GeneCards database (https://www.genecards. org/), we searched for relevant targets by entering "cancer/tumor". The Wayne plots of the active ingredient targets of PKVG rhizome phenolic acid analogs and cancer/tumor targets were plotted using the online software Venny 2.1.0 (http://www.liuxiaoyuyuan.cn/) to obtain PKVG rhizome phenolic acid analogs-cancer/tumor common targets, which were predicted to be the potential PKVG rhizome phenolic acid analogs treatment for cancer/tumor. The target was predicted to be a potential target for the treatment of cancer/tumor.

### Gene Ontology (GO) and Kyoto Encyclopedia of Genes and Genomes (KEGG) analyses

The common targets of PKVG rhizome phenolic acids and cancer/tumor were entered into the DAVID database (https://david.ncifcrf.gov/tools.jsp/) platform, and the species was limited to humans. GO enrichment analysis and KEGG pathway annotation analysis of potential targets of PKVG rhizome phenolic acids for cancer/tumor treatment were performed to screen important signaling pathways of phenolic acids in PKVG rhizomes for cancer/tumor treatment. The GO function enrichment histogram and KEGG signaling pathway enrichment bubble diagram were drawn in R software from the analysis results.

### Network construction and analysis

The compound-target-pathway network was constructed using Cytoscape 3.9.0. In the network, compounds, targets, and pathways were represented by nodes, and interactions between two nodes were represented by edges. In addition, the importance of each node in the networks was evaluated using a crucial topological parameter, namely degree. The topological properties were analyzed using the Network Analyzer plug-in for Cytoscape 3.9.0 to confirm the key components and targets, such as degree centrality (DC), betweenness centrality (BC), and closeness centrality (CC).

### Molecular docking techniques and visualization

The 3D structures of the active components of PKVG rhizome phenolic acid analogs were downloaded from the PubChem database (https://pubchem.ncbi.nlm.nih.gov/), and the receptor proteins corresponding to the key targets were downloaded from the RCSB PDB database (https://www.rcsb.org/). The sdf files were converted into the pdb file format using Discovery Studio 2019 software. PyMOL 4.3.0 software was used to separate the original ligand and protein structures and to dehydrate and remove the organics. Furthermore, AutoDockTools-1.5.7 software was used to process the proteins as follows: non-polar hydrogen was added, the Gasteiger charge was calculated, the AD4 type was assigned, and the flexible bonds of small molecules/ligands were set to be rotatable. Based on the original ligand coordinates, the docking box was adjusted to include all protein structures. Furthermore, the receptor protein was set to a semiflexible butt joint, and the Lamarckian genetic algorithm was selected. The docking results were obtained by running autogrid4 and autodock4; as a result, the binding energies were revealed. Finally, visualization and analysis were performed by PyMOL 4.3.0 software.

## Results

### Detection of phenolic acid metabolites and differential metabolite analysis of PKVG rhizomes

In order to identify and better understand the phenolic acid metabolites in PKVG rhizomes in more detail, we performed a broadly targeted metabolomic analysis of PKVG rhizomes. A total of 71 phenolic acids were identified (Table 1). As can be seen from Table 1, a total of 19 phenolic acids were detected in the positive ion mode and 52 in the negative ion mode. In addition, the top 5 phenolic acid compounds in PKVG rhizomes were 4-nitrophenol, diisobutyl phthalate, dibutyl phthalate, bis (2-ethylhexyl) phthalate, and diisooctyl phthalate, and all 5 compounds were detected in the positive ion mode.

**Table 1:** UPLC-MS/MS results of phenolic acid compounds in PKVG rhizome extracts.

Peak number	Index	Molecular weight (Da)	Formula	Ionization model	Compounds	Measured excimer ion peak (m/z)
1	pme2828	139.0269	C_6_H_5_NO_3_	+H	4-Nitrophenol	34271160.42
2	Lmxp011770	278.1518	C_16_H_22_O_4_	+H	Diisobutyl phthalate	13791107.00
3	Lmlp012720	278.1518	C_16_H_22_O_4_	+H	Dibutyl phthalate	13090094.91
4	Lmwp011196	390.277	C_24_H_38_O_4_	+H	Bis (2-ethylhexyl) phthalate	3976017.85
5	Lmmp010562	390.277	C_24_H_38_O_4_	+H	Diisooctyl Phthalate	3900247.07
6	pmn001367	316.0794	C_13_H_16_O_9_	-H	Protocatechuic acid-4-O-glucoside	2894640.82
7	pmb2871	316.0794	C_13_H_16_O_9_	-H	1-O-Gentisoyl-β-D-glucoside	1797950.01
8	Lmbp000728	120.0575	C_8_H_8_O	+H	(S)-2-Phenyloxirane	1089991.67
9	NK10264324	126.0317	C_6_H_6_O_3_	+H	Phloroglucinol; 1,3,5-Benzenetriol	1072580.24
10	pmb3142	300.0845	C_13_H_16_O_8_	-H	Salicylic acid-2-O-glucoside	1029527.16
11	Zmhn005413	336.0481	C_15_H_12_O_9_	-H	p-Dimeric galloyl methyl ester	1023436.25
12	pmb3107	360.1057	C_15_H_20_O_10_	-H	Glucosyringic Acid	900840.02
13	NK10253223	167.0582	C_8_H_9_NO_3_	+H	2-Amino-3-methoxybenzoic acid	650870.58
14	Lmyn000160	238.0689	C_8_H_14_O_8_	-H	Mucic acid Dimethyl Ester	487746.78
15	pmp001285	148.016	C_8_H_4_O_3_	+H	Phthalic anhydride	483762.15
16	Jmbp006554	388.1158	C_20_H_20_O_8_	+H	Ethyl Rosmarinate	432962.91
17	pmn001681	166.0994	C_10_H_14_O_2_	-H	1-(4-Methoxyphenyl)-1-propanol	387652.01
18	pmb2654	461.1533	C_19_H_27_NO_12_	-H	Anthranilate-1-O-Sophoroside	375673.28
19	pmb2497	198.0528	C_9_H_10_O_5_	-H	4-Hydroxy-3-methoxymandelate	357663.15
20	Zmhn001926	300.084	C_13_H_16_O_8_	-H	1-O-Salicyloyl-β-D-glucose	206817.47
21	pmn001518	332.0743	C_13_H_16_O_10_	-H	1-O-Galloyl-β-D-glucose	203521.01
22	Zmhn002422	356.1102	C_16_H_20_O_9_	-H	1-O-Feruloyl-β-D-glucose	201251.17
23	Hmqn000843	302.0998	C_13_H_18_O_8_	-H	Tachioside	192752.66
24	pmn001519	336.0481	C_15_H_12_O_9_	-H	Galloyl Methyl gallate	166514.75
25	mws0458	152.0474	C_8_H_8_O_3_	-H	Vanillin; 4-Hydroxy-3-Methoxybenzaldehyde	166341.69
26	Zmhn002301	326.0996	C_15_H_18_O_8_	-H	p-Coumaric acid-4-O-glucoside	154949.25
27	pmb0069	121.0528	C_7_H_7_NO	+H	Benzamide	152656.67
28	pme2598	168.042	C_8_H_8_O_4_	-H	3,4-Dihydroxybenzeneacetic acid	147197.50
29	pmn001419	326.1002	C_15_H_18_O_8_	-H	1-O-p-Coumaroyl-β-D-glucose	145298.75
30	Cmsp002787	226.0841	C_11_H_14_O_5_	+H	3,4’-Dihydroxy-3’,5’-dimethoxypropiophenone	135043.55
31	Hmhn003067	326.1002	C_15_H_18_O_8_	-H	Phenylpropionic acid-O-β-D-glucopyranoside	110752.27
32	MWSslk097	194.0579	C_10_H_10_O4	+H	Dimethyl phthalate	105455.28
33	Lmbn013410	206.1671	C_14_H_22_O	-H	2,4-Di-Tert-Butylphenol	101110.72
34	Lmln010063	206.1671	C_14_H_22_O	-H	2,6-Di-tert-butylphenol	98167.34
35	Lmtn002233	328.1158	C_15_H_20_O_8_	-H	Androsin	80932.86
36	MWSmce248	164.0473	C_9_H_8_O_3_	-H	3-Hydroxycinnamic Acid	80856.59
37	Zmhn002227	386.1208	C_17_H_22_O_10_	-H	4-O-Glucosyl-sinapate	66306.75
38	Wmmp000182	302.0063	C_14_H_6_O_8_	-H	ellag icacid	65552.68
39	pme3437	212.0685	C_10_H_12_O_5_	+H	Eudesmic acid (3,4,5-trimethoxybenzoic acid)	62081.79
40	Lmtn002324	432.1632	C_19_H_28_O_11_	-H	Benzyl-(2’’-O-glucosyl) glucoside	60778.40
41	pme0422	194.0579	C_10_H_10_O_4_	-H	Isoferulic Acid	59409.52
42	HJN003	386.1219	C_17_H_22_O_10_	-H	1-O-Sinapoyl-β-D-glucose	55351.41
43	mws0014	194.0579	C_10_H_10_O_4_	-H	Ferulic acid	55349.13
44	Lmyn003028	432.1632	C_19_H_28_O_11_	-H	Benzyl-β-gentiobioside	53797.74
45	MWSmce328	516.1268	C_25_H_24_O_12_	+H	Isochlorogenic acid C	51053.43
46	Yshj000011	332.1107	C_14_H_20_O_9_	+H	2,6-dimethoxy-4-hydroxyphenyl-1-O-beta-D-glucopyranoside	47820.04
47	Zmhn001793	342.0956	C_15_H_18_O_9_	-H	6-O-Caffeoyl-D-glucose	44859.41
48	mws0628	122.0368	C_7_H_6_O_2_	-H	4-Hydroxybenzaldehyde	43175.01
49	pmn001382	516.1268	C_25_H_24_O_12_	-H	Isochlorogenic acid A	43124.62
50	Li512115	516.1268	C_25_H_24_O_12_	-H	Isochlorogenic acid B	42291.21
51	Lman002731	342.0951	C_15_H_18_O_9_	-H	Grevilloside F	40605.37
52	pmn001315	458.1424	C_20_H_26_O_12_	-H	Regaloside	36128.30
53	pmb3061	500.153	C_22_H_28_O_13_	-H	5-O-p-Coumaroylquinic acid O-glucoside	33770.19
54	Lmmn001643	164.0473	C_9_H_8_O_3_	-H	2-Hydroxycinnamic acid	30989.15
55	Hmtn001302	300.0851	C_13_H_16_O_8_	-H	Glucosyloxybenzoic acid	28627.07
56	Lmzn001983	376.1369	C_16_H_24_O_10_	-H	D-Threo-guaiacylglycerol-7-O-β-D-glucoside	26420.43
57	pmb3064	500.153	C_22_H_28_O_13_	-H	3-O-p-Coumaroylquinic acid-O-glucoside	25851.64
58	pmn001690	328.1522	C_16_H_24_O_7_	-H	3-Hydroxy-4-isopropylbenzylalcohol-3-O-glucoside	24789.39
59	MWSslk066	168.0423	C_8_H_8_O_4_	+H	3-Hydroxy-4-methoxybenzoic acid; Isovanillic Acid	23066.94
60	Lmmn000774	344.1107	C_15_H_20_O_9_	-H	Dihydro caffeoyl glucose	18421.04
61	Lmjp003731	530.1424	C_26_H_26_O_12_	+H	3,4-O-Dicaffeoylquinic Acid Methyl Ester	15812.98
62	Lmjp003822	530.1424	C_26_H_26_O_12_	+H	3,5-O-Dicaffeoylquinic Acid Methyl Ester	15812.98
63	pme3443	208.0736	C_11_H_12_O_4_	-H	Sinapinaldehyde	15394.70
64	pmn001525	478.1475	C_23_H_26_O_11_	-H	3,5-Digalloylshikimic acid	9948.08
65	Jmwn002117	332.1107	C_14_H_20_O_9_	-H	2-(3,4-dihydroxyphenyl) ethanediol 1-O-β-D-glucopyranoside	9256.81
66	Lmmn001294	332.1107	C_14_H_20_O_9_	-H	Koaburaside	9256.81
67	pmn001511	286.1053	C_13_H_18_O_7_	-H	3-Hydroxy-5-Methylphenol-1-O-Glucoside	8358.38
68	mws0178	354.0951	C_16_H_18_O_9_	-H	Chlorogenic acid (3-O-Caffeoylquinic acid)	7336.56
69	Lmbn004847	180.0786	C_10_H_12_O_3_	-H	4-Methoxyphenylpropionic acid	6560.65
70	MWS1839	166.063	C_9_H_10_O_3_	-H	Ethylparaben	4314.38
71	MWS2070	180.0786	C_10_H_12_O_3_	-H	Propyl 4-hydroxybenzoate	3289.45

### Target recognition and disease mapping

**Fig 1:**
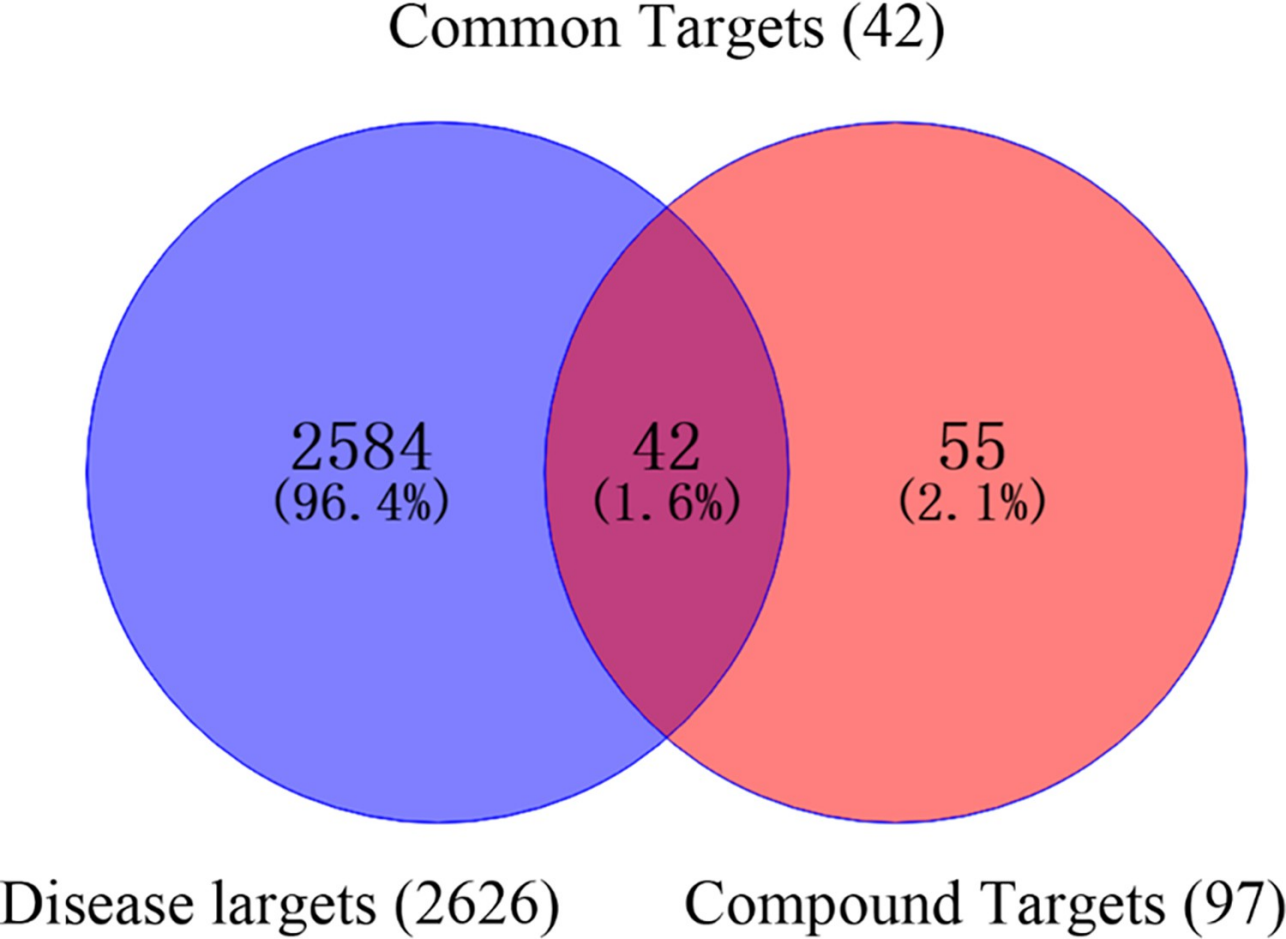
Venn diagram of antitumor activity of PKVG rhizome phenolic acid analogs.

Targeted identification of 71 constituents was performed using the TCMSP database. A total of 22 chemical components of TCM were identified. To further identify the key active ingredients, we used OB ≥ 10% and DL ≥ 0.1 as screening criteria. As a result, three compounds were identified among the 22 metabolites. These three phenolic acids were diisooctyl phthalate, androsin, and chlorogenic acid, and the analysis of their disease targets showed that 97 targets were obtained after removing duplicates. Then, 2626 targets were obtained from the Genecards database by entering "cancer/tumor" as a keyword. The analysis was performed using Venny 2.1.0 online software to identify common compound targets and disease targets (Fig 1). Forty-two targets were identified in terms of anti-cancer/tumor activity.

### GO and KEGG pathway enrichment analysis

**Fig 2:**
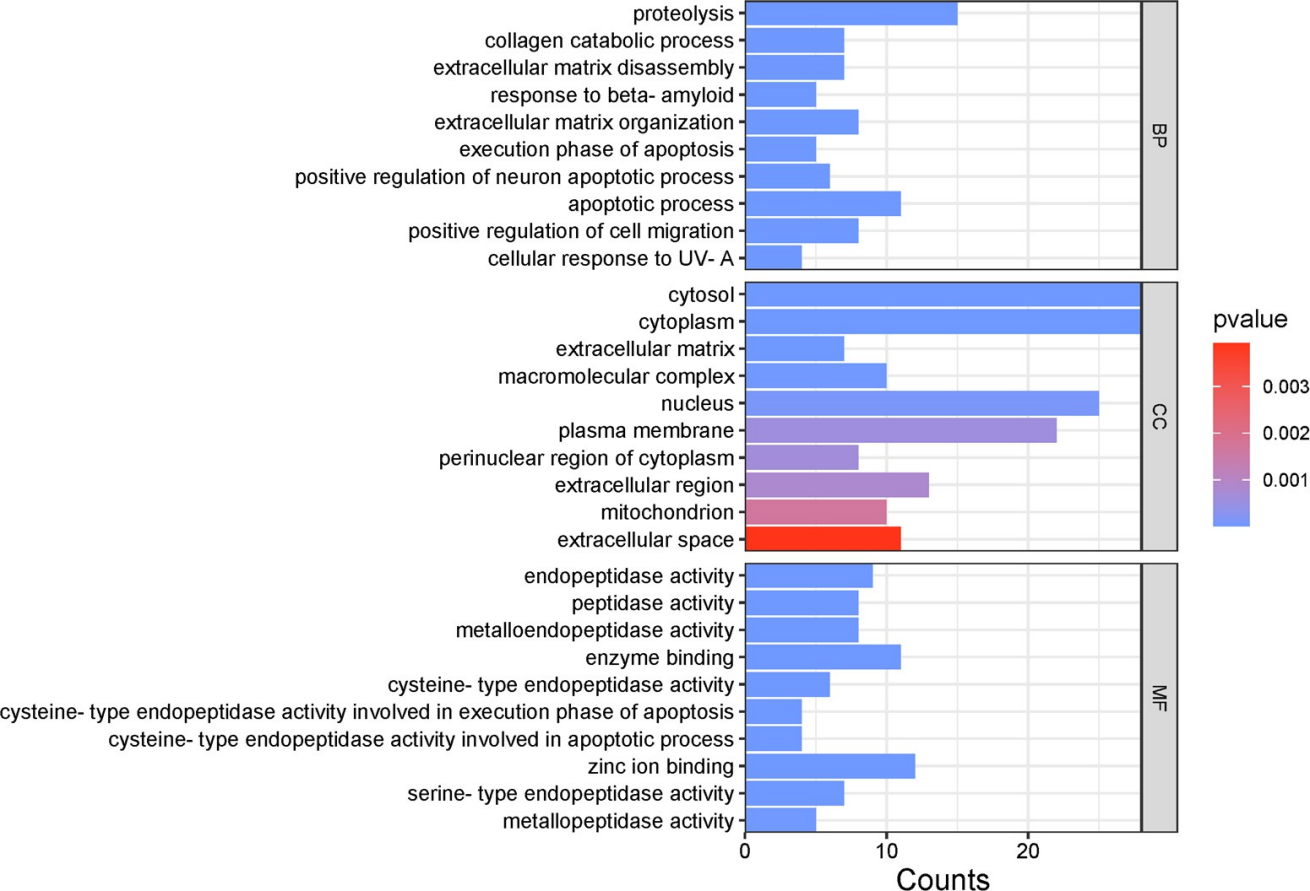
Results of GO pathway enrichment analysis for anti-cancer/tumor activity of phenolic acids from PKVG rhizomes.

The 42 common targets of phenolic acids and cancer/tumor were subjected to GO functional enrichment analysis using the DAVID platform. The GO functional enrichment analysis yielded 128 BP, which were filtered according to P-value and found to be mainly associated with proteolysis, collagen catabolic process, and extracellular matrix disassembly. The analysis yielded 21 CC entries, which were filtered according to P-value and were mainly associated with the cytosol, cytoplasm, and extracellular matrix. The analysis yielded 39 entries for MF, which were filtered based on P-value and were mainly associated with endopeptidase activity, peptidase activity, and metalloendopeptidase activity (Fig 2).

**Fig 3:**
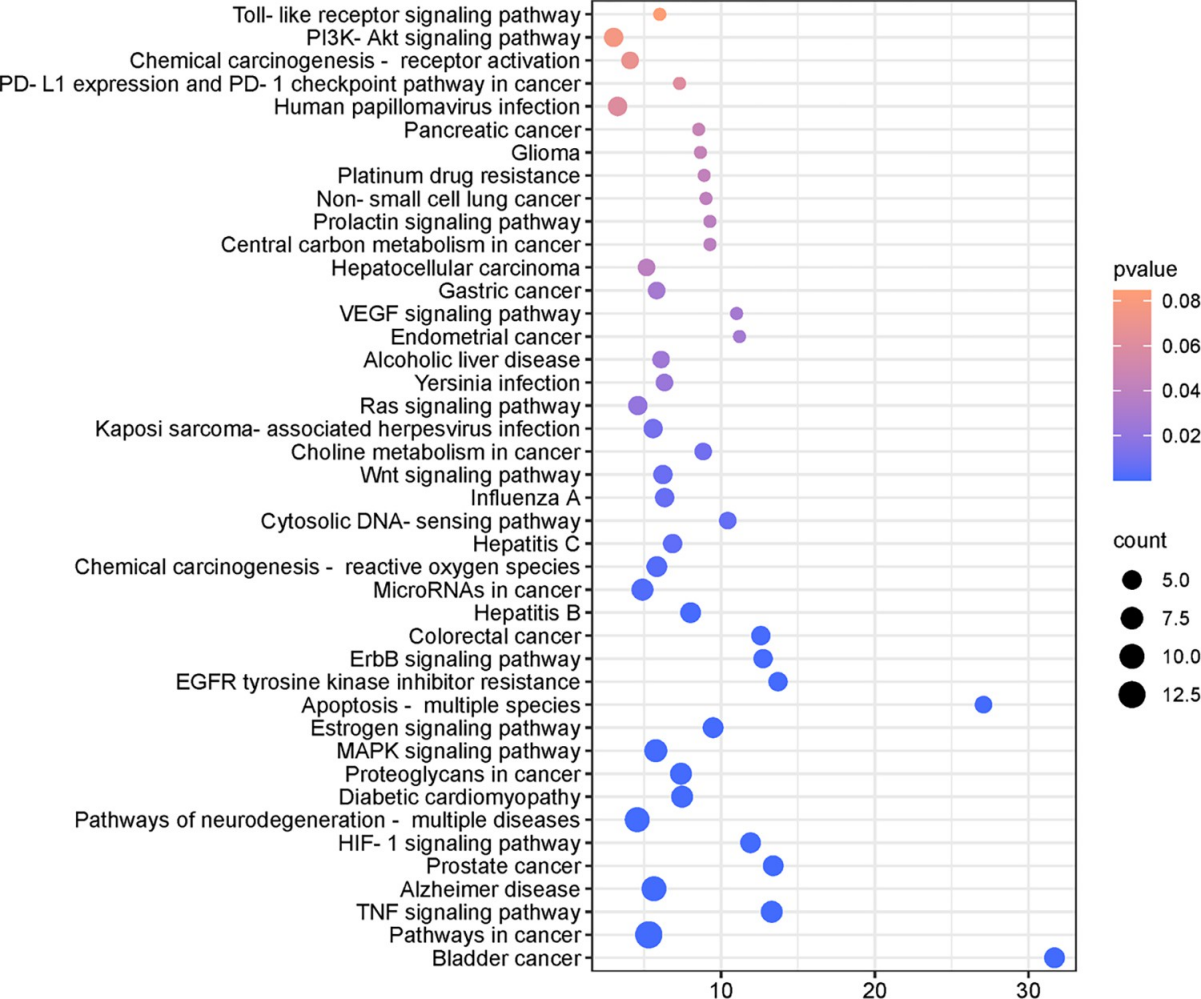
Results of KEGG pathway enrichment analysis for anti-cancer/tumor activity of phenolic acids from PKVG rhizomes. Bubble color and size correspond to the p-value and gene number enriched in the pathway.

Forty-two targets were enriched with 86 pathways associated with anti-cancer/tumor effects, of which 42 pathways were closely related to cancer/tumor. Fig 3 shows the results of the KEGG pathway analysis of anti-cancer/tumor effects of PKVG rhizomes. The results showed that pathways in cancer, pathways of neurodegeneration-multiple diseases, and the MAPK signaling pathway were the major pathways involved in the anti-cancer/tumor effects of PKVG rhizomes. Pathways in cancer had the largest number of Pathways in cancer have the largest number of anti-cancer/tumor-associated targets, including EGLN1, GSK3B, MMP1, MMP2, PRKCA, MMP9, EGFR, MAPK10, AR, CASP7, CASP8, CASP3, and MAPK1. Pathways of neurodegeneration-multiple diseases There are ten anti-cancer/tumor-associated targets, namely MAPK10, GSK3B, APP, CASP7, CASP8, CSNK2A1, HSPA5, CASP3, MAPK1, and PRKCA. The MAPK signaling pathway has eight targets, namely MAPK10, HSPA8, CASP3, KDR, MAPK1, PRKCA, EGFR, and CDC25B.

### Network construction and analysis

**Fig 4:**
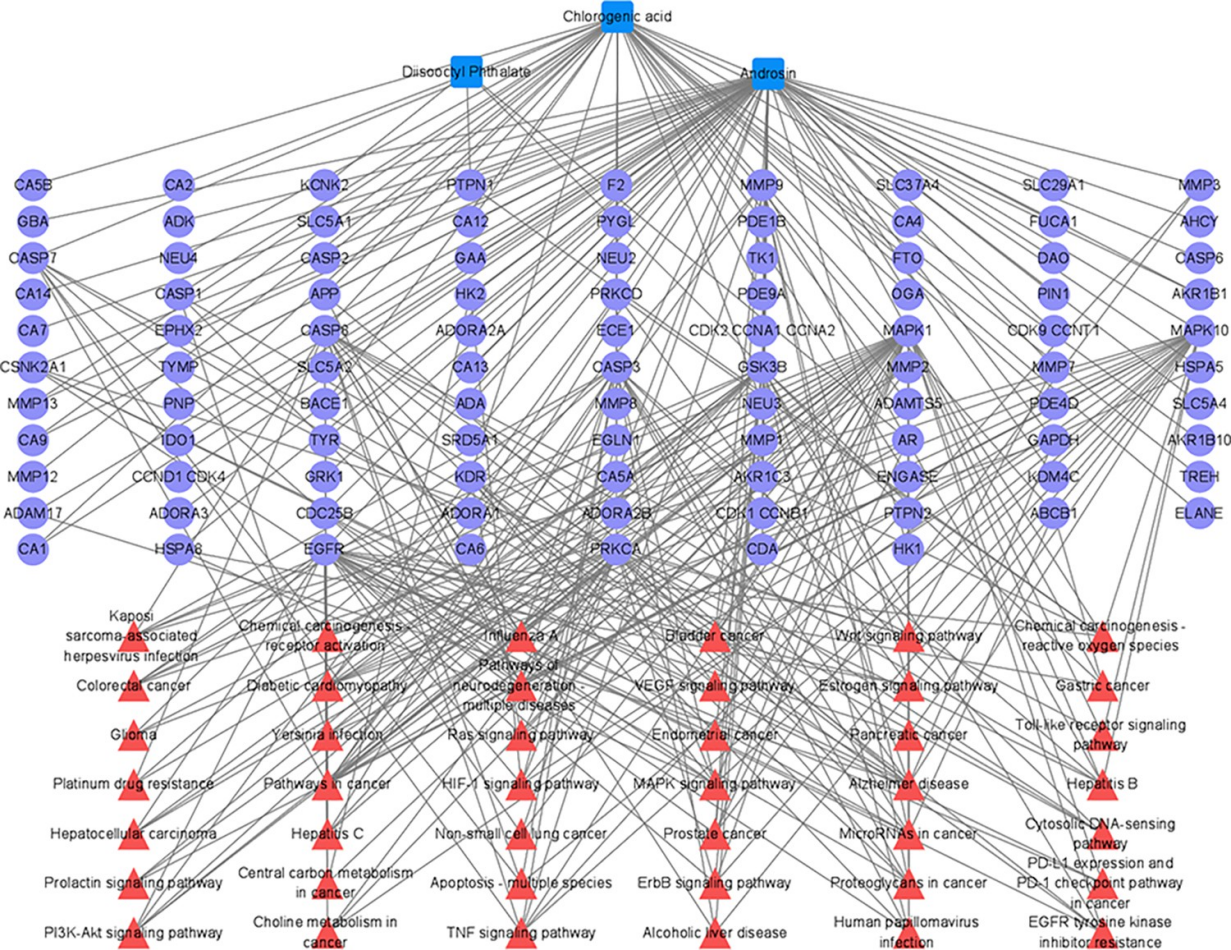
Compound-target-pathway network for anti-cancer/tumor activity of PKVG rhizome phenolic acid analogs. Blue squares represent active components in the PKVG rhizome, purple circles represent predicted targets, and red triangles represent associated pathways.

The PKVG rhizome compound-target-pathway network contains 142 nodes. In Fig 4, 3 compounds are shown in blue, 97 targets are shown in purple, and 42 pathways are shown in red. The core targets were mined using the Centiscape 2.2 plug-in in Cytoscape 3.9.1 software. BC, CC, and DC of target proteins were calculated using topological analysis. The selection criteria were set as follows: BC > 260.59, CC > 0.0025, and DC > 4.52. As shown in Table 2, we obtained 2 key compounds and 7 above-average values for the key targets. The four targets with the highest degree values among all targets were mitogen-activated protein kinase 1 (MAPK1; DC = 38, BC = 3016.51, CC = 0.0034), epidermal growth factor receptor (EGFR; DC = 27, BC = 1552.31, CC = 0.0031), and mitogen-activated protein kinase 10 (MAPK10; DC = 21, BC = 1177.56, CC = 0.0030) associated with androsin. While classical protein kinase C alpha type (PRKCA; DC = 21, BC = 1129.64, CC = 0.0027) is associated with chlorogenic acid.

**Table 2:** Network analysis results of key active ingredients, key targets and key pathways for anti-cancer/tumor activity of PKVG rhizome phenolic acid analogs.

No.	Name	Betweenness Centrality (BC)	Closeness Centrality (CC)	Degree Centrality (DC)
1	Androsin	11308.37	0.0039	62
2	Chlorogenic acid	6314.03	0.0031	38
3	MAPK1	3016.51	0.0034	38
4	EGFR	1552.31	0.0031	27
5	PRKCA	1129.64	0.0027	21
6	MAPK10	1177.56	0.0030	21
7	GSK3B	1020.74	0.0030	20
8	CASP3	789.40	0.0026	18
9	CASP8	599.82	0.0026	15
10	MMP9	383.00	0.0028	10
11	Pathways in cancer	769.10	0.0028	13
12	Alzheimer disease	342.20	0.0027	10
13	Pathways of neurodegeneration—multiple diseases	325.84	0.0027	10

### Molecular docking results

**Fig 5:**
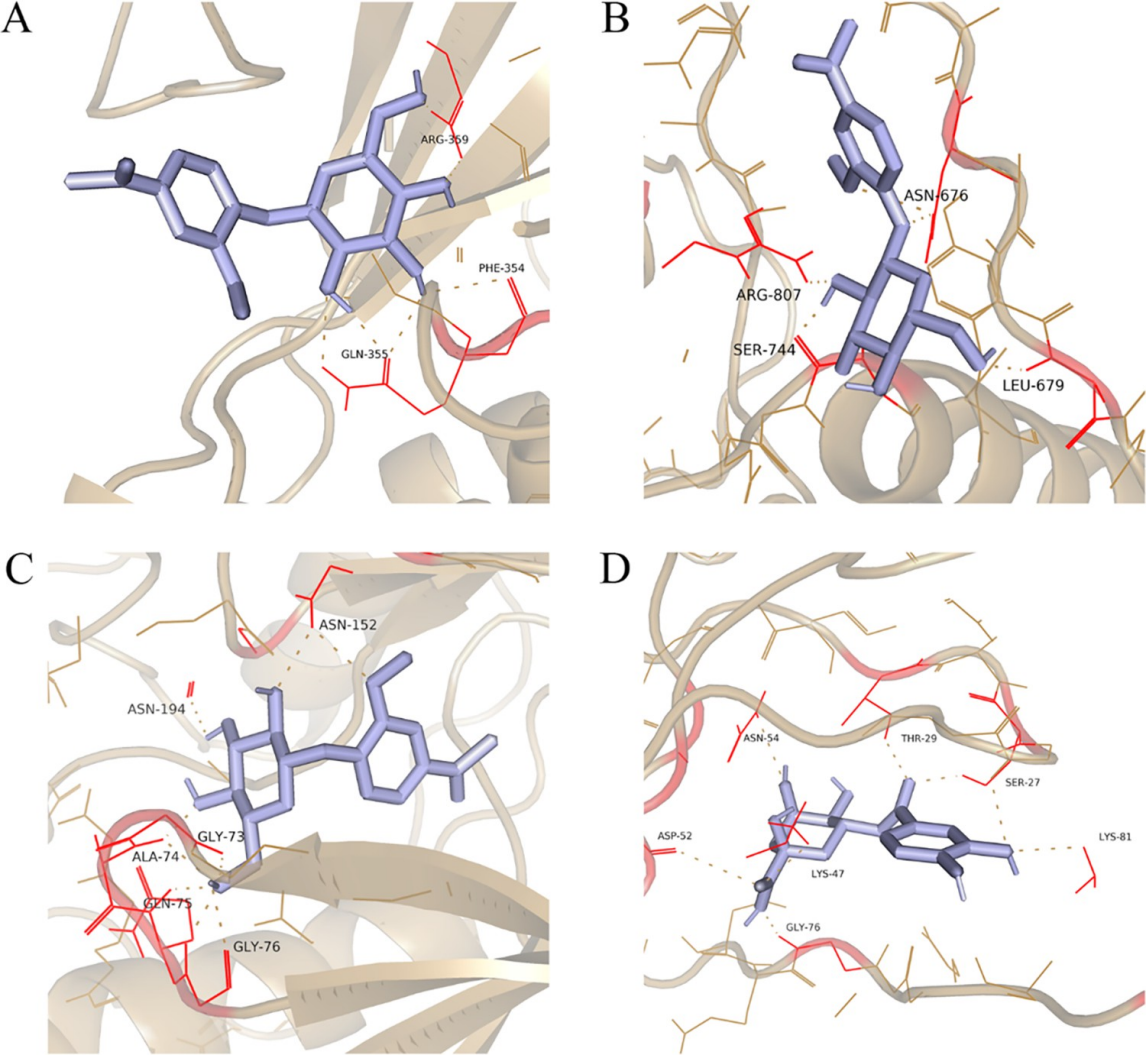
Interaction graphics between the compounds and targets. (A) Androsin-MAPK1, (B) Androsin-EGFR, (C) Androsin-MAPK10, (D) Chlorogenic acid-PRKCA.

The key compounds androsin and chlorogenic acid were molecularly docked to the four core targets with the highest degree of centrality: MAPK1, EGFR, MAPK10, and PRKCA. The binding energies are shown in Table 3. During molecular docking, the Androsin-MAPK1 docking parameter center (x, y, z) was (-4.433, 8.76, 47.404), the Androsin-EGFR docking parameter center (x, y, z) was (23.581, 9.73, 58.824), the Androsin-MAPK10 docking parameter center (x, y, z) was (9.249, 19.879, 23.573), and the Chlorogenic acid-PRKCA docking parameter center (x, y, z) was (5.254, 2.591, -0.453). All sizes (x, y, z) were (126, 126, 126). In order to evaluate the binding ability of key compounds and key targets, we used the empirical threshold (-5.0 kcal/mol) mentioned in the literature as an evaluation criterion, and if the docking binding energy was below the threshold, it indicated that the target had a strong binding ability to the compounds. The results showed that the binding ability of these key compounds to the four key targets was above the empirical threshold. We further analyzed the compound-target interactions (Fig 5). The results showed that Androsin had hydrogen bonds with all three targets (MAPK1, EGFR, and MAPK10), such as 3 hydrogen bonds in androsin-MAPK1, 4 hydrogen bonds in androsin-EGFR, androsin-MAPK10 has 6 hydrogen bonds, and chlorogenic acid-PRKCA has 7 hydrogen bonds. The presence of these types of bonds is the main reason why the binding energies of the complexes are all below the threshold. These results are also consistent with the results of the KEGG pathway enrichment analysis.

**Table 3:** Energy docking results of key targets and key phenolic acid compounds from PKVG rhizome.

Protein name	Gene name	Ligand name	Binding energy (kcal/mol)
MAP kinase ERK2	MAPK1	Androsin	-6.1
Epidermal growth factor receptor erbB1	EGFR		-5.8
c-Jun N-terminal kinase 3	MAPK10		-7.8
Protein kinase C alpha	PRKCA	Chlorogenic acid	-6.8

## Discussion

With the growing interest in the study of traditional Chinese herbs, many bioactive compounds have shown great pharmacological, biological, and medicinal value, including potential anticancer activities [17]. Cancer is currently a difficult challenge for mankind to overcome, and its occurrence is a complex process involving multiple steps in the transformation of normal cells into uncontrolled, proliferating cancer cells, a process commonly referred to as carcinogenesis or tumorigenesis [18]. In recent years, the active ingredients in many Chinese herbal medicines have been investigated using network pharmacology and molecular docking, and many targets for cancer/tumor treatment have been identified. For example, network pharmacology prediction and molecular docking of active ingredients in *Salvia miltiorrhiza* have been performed, and SRC, IL6, and INS were found to be associated with colorectal cancer [19]. Network pharmacological prediction and molecular docking of active ingredients in *Astragalus membranaceus* revealed that 1,7-dihydroxy-3,9-dimethoxy pterocarpene and isoflavanone may be the main active ingredients in the treatment of glioma [20]. Network pharmacological prediction and molecular docking of active ingredients from *Angelica dahurica* revealed that sen-byakangelicol, beta-sitosterol, and prangenin have high therapeutic osteosarcoma patients and improved osteosarcoma patients’ five-year survival rates [21]. All of the above studies demonstrate the important role of network pharmacology prediction and molecular docking technology in the study of cancer therapy.

In this study, we identified 71 phenolic acids in PKVG rhizomes using broadly targeted metabolomics techniques. Three active ingredients (diisooctyl phthalate, androsin, and chlorogenic acid) were obtained by searching through the TCMSP database. These constituents with good medicinal properties are the material basis for the treatment of cancer/tumor with phenolic acids from PKVG rhizomes. There are 42 intersecting targets of cancer/tumor with PKVG phenolic acid-based active ingredients, and the top four DC were MAPK1, EGFR, MAPK10, and PRKCA by network analysis. Among them, the expression of MAPK1 inhibits the proliferation of rectal cancer cells [22]. CSIG-03. EGFR ligand in EGFR-amplified glioblastoma was able to activate the tumor suppressor of EGFR, which resulted in the conversion of EGFR oncogenes to oncogenes [23]. The tumor suppressor miR-335-5p was able to inhibit gastric cancer by targeting MAPK10 [24]. However, upregulation of PRKCA may be an unfavorable factor that leads to lung adenocarcinoma [25]. Therefore, it is important to explore the relationship between these target genes and the active components of PKVG phenolic acids to understand the mechanism of PKVG treatment of cancer/tumor.

GO functional enrichment and KEGG pathway analysis were performed on PKVG and cancer/tumor common target genes. It was found that GO enrichment analysis predicted that the active ingredients of luteolin might exert their cancer/tumor therapeutic effects through biological processes such as proteolysis, the collagen catabolic process, and extracellular matrix disassembly of the active ingredients of luteolin. The above study suggests that multiple biological processes mediating and participating are the key to the anti-cancer/tumor effects of herbal PKVG. In KEGG enrichment analysis, inhibition of the PI3K-Akt signaling pathway is essential to inhibit cancer/tumor development. Aberrant activation of this signaling pathway is one of the most frequent events in human cancer and serves to disconnect the control of cell growth, survival and metabolism from exogenous growth stimuli [26, 27]. MAPK signaling pathway may be involved in the carcinogenesis and development of human meningiomas by binding to HER-2 [28], and inhibition of MAPK signaling pathway inhibition also suppresses glioma development [29]. Blocking the HIF-1 signaling pathway can prevent and treat cancer [30]. Moreover, the KEGG-enriched IL-17 signaling pathway [31] and TNF signaling pathway [32], are closely associated with cancer development.

We identified androsin and chlorogenic acid as the key herbal active ingredients in the rhizomes of PKVG through the TCMSP database. Previous studies have shown that androsin treatment not only reduces lipid levels in mouse hepatocytes and serum but also reduces HFrD-induced alanine aminotransferase (ALT), aspartate transaminase (AST), and cholesterol, resulting in the improvement of liver health in mice [33]. In addition, androsin has defensive effects against allergens and PAF-induced bronchial obstruction in guinea pigs [34]. However, so far, no study has demonstrated the inhibitory effect of androsin on cancer/tumor. However, in the present study, we found that androsin may have some therapeutic effects on cancer/tumor after medicinal target screening and molecular docking. Chlorogenic acid can regulate the glycolysis of tumor cells under hypoxic conditions [35], and its loaded chitosan nanoparticles also have tumor-preventive and antioxidant efficacy in experimental skin carcinogenesis [36]. The above result is similar to the predicted results of the present study.

## Conclusion

The phenolic acid chemical constituents in PKVG rhizomes were qualitatively and quantitatively analyzed using broadly targeted metabolomics techniques. From this, we identified 71 compounds; we then used a network pharmacology approach for target identification, pathway analysis, and network construction. Using this approach, the material basis and molecular mechanism of the anti-cancer/tumor effects of phenolic acids in PKVG rhizomes were explained for the first time to the best of our knowledge. Two key active components and eight key targets were obtained, and 42 major pathways were identified by KEGG pathway analysis. This finding reflects the multi-component, multi-target, and multi-pathway characteristics of traditional Chinese medicine.
