# Supplementary material for: Metabolomics and network pharmacology-based identification of phenolic acids in Polygonatum kingianum var. grandifolium rhizomes as anti-cancer/Tumor active ingredients
Source: PLoS One. 2024 Dec 17;19(12):e0315857. doi: 10.1371/journal.pone.0315857 (PMC11651577; doi:10.1371/journal.pone.0315857)
Supplement: Attachment — Submitted filename: Response to Reviewers.docx [file pone.0315857.s001.docx]

**Reply to Reviewers’ comments**

Dear Editor,

Thank you very much for kindly providing us with the opportunity to revise our manuscript entitled " **Metabolomics and network pharmacology-based identification of phenolic acids in *Polygonatum kingianum* var. *grandifolium* rhizomes as anti-Cancer/Tumor active ingredients** ". The reviewers’ comments are all valuable and very helpful for revising and improving our paper. The revised sections are highlighted in the revision. The main corrections in the paper and the response to the reviewer’s comments are as follows:

**Response to Reviewers:**

**Reviewer #1:**

1. Methods are not described in enough detail, and finally, Formatting inconsistencies throughout the manuscript, such as citation styles and section headings, could detract from its overall professionalism. Addressing these section-wise issues is crucial for enhancing the manuscript's quality and its chances of acceptance for publication. In the other sections its been explained properly and the funding details if given in detail it could be better

**Response:** We sincerely thank **Reviewer #1** for the meticulous comment. Firstly, we provided a more detailed description of the methodology used in the article. Please refer to lines 111-186 of the revised manuscript for details. Secondly, we thoroughly revised the manuscript's chapter titles and citation formats to ensure consistency. Finally, we have added detailed funding information in lines 365–371 of the article. Thank you for your valuable comments.

**Reviewer #2:**

1. The manuscript technically sound, and the data support the conclusions. The conclusions drawn appropriately based on the data presented. The manuscript presented in clear and comprehensible fashion and written in standard English.

**Response:** We sincerely thank **Reviewer #2** for the meticulous comment. To ensure its accuracy, we have made every effort to revise the article's grammar content. Thank you for your valuable comments.
