# Supplementary material for: Metabolomics and network pharmacology-based identification of phenolic acids in Polygonatum kingianum var. grandifolium rhizomes as anti-cancer/Tumor active ingredients
Source: PLoS One. 2024 Dec 17;19(12):e0315857. doi: 10.1371/journal.pone.0315857 (PMC11651577; doi:10.1371/journal.pone.0315857)
Supplement: Attachment — Submitted filename: Response to Reviewers.docx [file pone.0315857.s002.docx]

**Reply to Reviewers’ comments**

Dear Editor,

Thank you very much for kindly providing us with the opportunity to revise our manuscript entitled " **Metabolomics and network pharmacology-based identification of phenolic acids in *Polygonatum kingianum* var. *grandifolium* rhizomes as anti-Cancer/Tumor active ingredients** ". The reviewers’ comments are all valuable and very helpful for revising and improving our paper. The revised sections are highlighted in the revision. The main corrections in the paper and the response to the reviewer’s comments are as follows:

**Response to Reviewers:**

**Reviewer #3:**

1. Authors are requested to double-check the grammar of the entire text.

**Response:** We have done our best to correct the grammatical issues in the article.

2. How to identify metabolites needs to be clearly stated in the method.

**Response:** We added how to identify metabolites in the Materials and Methods section. See lines 112-132 of the revised draft for details.

3. What is the basis for screening criteria OB ≥ 5% and DL ≥ 0.14?

**Response:** Although the commonly used criteria are OB ≥ 30% and DL ≥ 0.1, this is not absolute. In a study on astragalus-albizia medicine at the First Clinical Hospital of Shandong University of Traditional Chinese Medicine, researchers used OB ≥ 10% and DL ≥ 0.1 as the screening criteria and found that IL-1β is a potentially key mechanism for the treatment of chronic obstructive pulmonary disease (COPD). Therefore, we also used this criterion for the Chinese herb *Polygonatum kingianum* var. *grandifolium*. We have checked this section and cited the relevant literature in the manuscript. See line 142 and reference 16 of the revised manuscript for details.

4. The topic of this work is too broad; it is recommended to choose one type of cancer or tumor for research.

**Response:** The aim of our study is to get the active components and targets of *Polygonatum kingianum* var. *grandifolium* phenolic acid analogs related to anti-cancer or tumors so as to lay the foundation for future research in our group. Therefore, our screening scope is broad. Other members of our group are conducting network pharmacological studies and animal studies on specific cancers or tumors based on existing research data. Therefore, we would like to publish this research article that analyzes a wide range of studies.

5. Please add molecular docking coordinates and BOX information used in present work.

**Response:** We have added the relevant information. See lines 270-275 of the revised draft for details.

6. “C18” 18 Subscript required, please verify similar specialized expressions.

**Response:** We have scrutinized and revised the contents of the manuscript. See Table 1 of the revised manuscript for details.

7. The discussion section also needs to go deeper in response to the results.

**Response:** We provide a more in-depth discussion of the results section. See the discussion section of the revised draft for more details.

**Reviewer #4:**

1. Legends are left with poor specifications.

**Response:** We have revised the content of the legend. See the revised draft for details.

If you have any questions about our manuscript, please feel free to contact us and we will get back to you as soon as possible.
